# Annotations

**Published:** 1888-05-05

**Authors:** 


### ANNOTATIONS.

**Will Workmen Give?**

"FIFTEEN thousand pounds," said Mr. Burdett-Coutts at The Hospitals Association on Wednesday, "is the amount contributed annually by the Glasgow workmen to the Glasgow Royal Infirmary." Fifteen thousand pounds a year to one hospital! There are in Glasgow at least three large general hospitals beside special hospitals and other medical charities of various kinds. In Leeds the sum raised annually by working-men is also counted by thousands, and in Sunderland the amount is very large. London can swallow up Glasgow, Leeds, and Sunderland together, six times over, and yet eleven thousand pounds, the amount raised by the Hospital Saturday Fund, represents the larger proportion of what London workmen do for all their hospitals. Here is a fine opportunity for a diatribe. But we must put the saddle on the right horse. Who is to blame for the sorry figure the London workmen cut in comparison with their Scotch and Yorkshire confrères? Is it the workmen themselves, or is it the organisations by means of which their contributions are collected? There are such things as undeveloped resources; gold mines, or mines of copper, tin, or coal which represent fabulous wealth, but which the unskilled farmer walks over day by day without suspecting. Are the London workshops and the factories of other large English towns mines of unexplored wealth waiting for the enterprise of the hospital manager? It is impossible to say. One thing, however, is certain, that if mines of any kind in any part of the world are so much as suspected by British mining engineers and British capitalists, there is not much time lost before at least an exploration is made. It seems to us that responsible hospital managers have not explored London sufficiently for the gold which they so constantly need. If they have, and London workmen cannot raise more than from eleven to twelve or fourteen thousand pounds annually for all their hospitals, whilst a portion of the Glasgow workers contribute fifteen thousand annually for one, there must be something radically wrong either with the disposition or the resources of London operatives. We are inclined to think, however, that the fault lies with the various hospital engineers who do the mining operations in London. Perhaps they do not understand their business thoroughly! Perhaps they do not carry it on with energy! We should like very much to know the methods employed at Glasgow in the production of such splendid results. Scotch people get the credit of being close-fisted; but it is clear that some Scotchmen—or are they women?—have very effective methods of opening the fists of others. It might render a great service to London and other places if the Glasgow organisers would tell the world how they open their oysters. To alter the words of Carlyle a little—"Almost do some of the London hospitals decease, so obstinately do *their* oysters remain shut."

**Can Workmen Pay?**

THE Hospitals Association discussed at St. Thomas's Hospital on Wednesday a rather stale theme in a by no means stale fashion. The pay system was on the *tapis*, and Mr. Burdett-Coutts, M.P., occupied the place of orator. The paper was more than good. It was full of well-chosen facts, and as strong in logic as in figures. Mr. Burdett-Coutts is a thoroughly-convinced advocate of a judicious pay system. We do not propose to enter at length upon the question now, because the discussion of the paper at St. Thomas's was so keen and full of interest that it was resolved to continue it in June. When all has been said which can be said by the champions of both sides, we shall hope to gather up some practical conclusions. In the meantime we may point out that the reader of the paper took up a position so sound in its logic, so buttressed round with facts of all kinds, and withal held it so intelligently and defended it with such unmistakable skill, that the opponents will have to furbish up their best artillery if they are to dislodge him. The speaking was almost all on one side, and that side antagonistic to the pay system. But it cannot be said that the speeches were equal to the occasion, or to the paper which had been read. There was no attempt on the part of most of the speakers to deal with broad principles. Each of them seemed to think he had done enough when he had related his personal experience of his own hospital, and wound up his delivery with a *non possumus*. But that kind of thing will not do. Here is a whole hospital system on its trial; a system which is responsible for the expenditure of several millions sterling annually; a system which is boldly and intelligently assailed; and the only reply its defenders have is "We can't help it." It is certain that the besieged could make a better defence if they would take a little more trouble in making themselves master of the question. It is not creditable to hospital physicians and managers to show such a feeble grasp of economic principles, and such tenacity about small and trifling details. The pay system is either good or it is not good; it is either adapted to hospitals as a whole, or it is not so adapted. It is either elastic enough to meet every case, or it is not; it is either indispensable, or it is not. The last refuge of the destitute in debate is the *non possumus;* and the moment the defender is driven into that position the assailant considers the victory won—at any rate the argumentative victory. When the strife is over in the arena of debate, the practical downfall of the citadel is only a matter of time. If the defenders of the free system are to hold their own they must look to their artillery.

**Pasteurism and Hydrophobia.**

PROFESSORS BILLROTH and Virchow are still opposed to Pasteurism. This is significant. It is expected that Billroth will shortly publish his reasons for objecting to the spending of public money on further experiments; and Virchow is said to be decidedly against such experiments, and "considers them dangerous and not founded upon scientific principles." We have always maintained, in opposition to many eminent British scientists, that the only proper verdict which the evidence as yet justifies in the case of Pasteur's experiments is the Scotch one of "Not proven." So far as abstract reasoning may help a right decision, it seems to us, as it does to Virchow, that the inoculation treatment for hydrophobia, as practised by Pasteur, is not founded on scientific principles. For what are the facts of the case? A man bitten by a rabid dog is presumably inoculated by the bite with a virus of great intensity. The Paris treatment proposes to cure him by inoculating him with a virus of much feebler intensity, at some time later than the original inoculation. The object of the second inoculation is, by means of the virus then introduced, to exhaust the pabulum on which the first virus depends for continued life and the dangerous multiplication of its specific microbes. But what does this mean? It means that the virus of weak intensity has to act with so much greater rapidity that it will, so to speak, overtake and pass by the original virus in its attack upon the tissues, and by its greater rapidity of action clear the country of all means of subsistence before the original and stronger virus comes up. When it is remembered that the two viruses are the same in kind, although the one is weaker than the other, it must be admitted that there is likely to be a certain similarity in their effects. But it can hardly be denied that if one of them is likely to act more promptly and energetically than the other, it will not surely be the weak virus, which has been started off later in the race, but the strong one, which has not only the advantage of its greatly-added intensity, but the very evident advantage of a definite number of hours' start. Arguing out the probabilities, it would seem decidedly that they are entirely against the soundness of Pasteur's conclusions. But scientific men prefer experiment to ratiocination alone, and therein they are right. If Pasteur's facts had been conclusive, we need not have troubled about his theories. The facts, however, seem to us to fail entirely in establishing the value of his treatment. Our British scientists, who pronounced in his favour, were, to say the least, premature, and did not act in accordance with the best traditions of British medicine and science. It seems to us, as we have before said, that on the evidence available the verdict of "Not proven" is the only one which satisfies dispassionate reason.

